# Relative validity of a brief dietary survey to assess food intake and adherence to national dietary guidelines among Sri Lankan adults

**DOI:** 10.1186/s40795-020-00391-2

**Published:** 2020-11-26

**Authors:** J. Renzella, S. Fernando, B. Kalupahana, N. Townsend, M. Rayner, K. Wickramasinghe, P. Katulanda, P. Scarborough

**Affiliations:** 1 Centre on Population Approaches for NCD Prevention, Nuffield Department of Population Health, University of Oxford, Oxford, UK; 2 Sri Jayewardenepura General Hospital and Post Graduate Training Centre, Colombo, Sri Lanka; 3 Tianjin Medical University, Tianjin, China; 4 Department of Health, University of Bath, Bath, UK; 5 WHO European Office for Prevention and Control of Non-communicable Diseases, WHO Regional Office for Europe, Moscow, Russia; 6 Department of Clinical Medicine, Faculty of Medicine, University of Colombo, Colombo, Sri Lanka; 7 NIHR Biomedical Research Centre at Oxford and Centre on Population Approaches for NCD Prevention, Nuffield Department of Population Health, University of Oxford, Oxford, UK

**Keywords:** Diet, Survey, Validation study, Adults, Sri Lanka, 24-h dietary recall, Food based dietary guidelines

## Abstract

**Background:**

Suboptimal diet is the leading cause of global morbidity and mortality. Addressing this problem requires context-specific solutions informed by context-specific data collected by context-specific tools. This study aimed to assess the relative validity of a newly developed brief dietary survey to estimate food intake and adherence to the Food Based Dietary Guidelines for Sri Lankans.

**Methods:**

Between December 2018 and February 2019, we interviewed 94 Sri Lankan adults living in Colombo (Western Province), Kalutara (Western Province), and Trincomalee (Eastern Province). We assessed the relative validity of the Sri Lankan Brief Dietary Survey (SLBDS) with Wilcoxon rank-sum tests, Spearman’s Rho correlation coefficients, Bland–Altman plots, and Cohen’s kappa tests using a 24-h Dietary Recall (24DR) as reference.

**Results:**

Ninety-four adults (40.7 years ±12.6; 66% female) completed both surveys during the same interview. With the exception of ‘Fish, pulses, meat and eggs’ food group median intake, which was underestimated by the SLBDS compared to the 24DR, there was no strong evidence of difference between median intakes reported by the two methods. Correlation coefficients were highest for ‘Milk and dairy products’ (0.84) at the food group level and for ‘dosa’, ‘hoppers’, ‘milk rice’, and ‘dried fish’ (1.00) among individual food and beverages. Visual exploration of Bland-Altman plots showed acceptable agreement between the SLBDS and 24DR, with the SLBDS tending to overestimate consumption as the number of servings of ‘Rice, bread, other cereals and yams’ and ‘Vegetables’ consumed increased and slightly underestimate consumption as the number of servings of ‘Fish, pulses, meat and eggs’, ‘Milk and dairy products’, and ‘Nuts’ increased. Kappa values ranged from from 0.59 (95% CI: 0.32–0.86) for ‘Vegetables’ to 0.81 (95% CI: 0.66–0.96) for ‘Fruit’ indicating a moderate to strong level of agreement.

**Conclusions:**

Having been developed for and relatively validated with the study population in question, our study shows that the SLBDS can be used as a fit for purpose research tool. Additional research is needed to assess SLBDS test-retest reliability and to validate further the reporting of salt, oil, and coconut intake.

**Supplementary Information:**

**Supplementary information** accompanies this paper at 10.1186/s40795-020-00391-2.

## Background

Unhealthy diet is the leading modifiable risk factor for the development of noncommunicable diseases (NCDs) [1]. Despite the promising global progress made in preventing and controlling NCDs in the last decades, they remain the world’s leading causes of death. This is in part due to a combination of unsustained and ineffective action mounted against key modifiable risk factors and the many ‘data blind spots’ that result from inadequate and infrequent data collection [2]. Substantial reductions in NCD morbidity and mortality therefore require increased investment in evidence-informed healthy diet promotion [3]. This necessitates accurate and timely dietary data collection to identify trends, develop appropriate solutions, prioritise resource allocation, and evaluate intervention effectiveness. Dietary intake, however, is notoriously difficult to capture accurately in population-based studies, and the complex and burdensome nature of ‘gold standards’ can limit their context suitability and sustainability [4, 5].

While diet may be the single most important risk factor for NCDs, it is by no means the only one. Embedding dietary intake measurements within multi-topic, multi-component national NCD risk factor surveys that collect behavioural, physical, and biochemical measurements in an attempt to avoid survey fatigue is therefore a popular surveillance approach [6]. The WHO STEPwise Surveillance (STEPS) methodology, for example, recommends Member States undertake multi-topic, multi-component STEPS surveys every 3–5 years – a schedule that has proved challenging in resource-constrained settings [6]. Supplementing these hugely important surveillance systems with short and easy to administer surveys that facilitate the periodic collection of good-quality data therefore has appeal – especially in resource-constrained settings where data collectors may receive minimal training [7]. The availability of validated brief dietary assessment tools, however, is limited, with many focussing on few, select food groups (for example, fruit and vegetable intake) [8].

Where data are being collected, linking these data to action – the ultimate goal of dietary surveillance – is not always realised [9]. Context-specific research tools designed to inform context-appropriate interventions may help to close this knowledge to action gap. This, of course, must include an understanding of contextual gender differences in dietary intake informed by self-report methods that are equally valid for collecting dietary intake data from women and men – an area of research that is currently underserved [10].

Country-specific and evidence-informed national dietary guidelines provide a convenient and logical structure to inform the development of a short dietary assessment tool; measuring recommendation compliance generates research outcomes that can be used to assess diet-disease associations and inform and evaluate national nutrition policy and public health interventions [11]. Often developed and endorsed by multiple government sectors (for example, the Ministry of Health, the Ministry of Education, and the Ministry of Agriculture), these documents and the activities born from them also provide a ready-made rallying point for the multisectoral action required to effectively tackle NCDs [3].

### Brief dietary survey for Sri Lankan adults

The rise in diet-related NCDs in Sri Lanka increases attention and importance on nutritional surveillance research [12–14]; the seven-year gap between the most recent Sri Lankan STEPS surveys (conducted in 2008 and 2015) highlights the need for contextually-validated, efficient, and easy to use dietary surveillance supplements [15]. Other methods currently used in the Sri Lankan NCD research context make important contributions but have practical constraints. The contextually validated Food Frequency Questionnaire for Sri Lankan Adults (SLFFQ) incurs both high memory and analysis burden [16] and the ‘international’ 24-h Dietary Recall (24DR) requires up to one hour to complete and a highly trained interviewer to administer. The Sri Lankan Brief Dietary Survey (SLBDS) (Fig. S1) was therefore developed with the following considerations in mind: 1) context suitability; 2) low administration burden for participants and researchers; 3) rapid and straightforward analysis; 4) suitability for repeat assessments in large-scale follow-up studies; 5) reduced respondent fatigue in multi-risk factor surveys; and 6) ability to provide useful health-related information to inform population-level policy and interventions.

In this study, we aimed to assess the relative validity of this newly developed brief dietary survey to estimate food intake and adherence to the Food Based Dietary Guidelines for Sri Lankans (SLFBDGs), using a 24DR [17] as reference. SLBDS content and structure are based on the SLFBDGs [18]. The core food groups outlined in the SLFBDGs and queried by the SLBDS are (1) ‘Rice, bread, other cereals and yams’; (2) ‘Fruit’; (3) ‘Vegetables’; (4) ‘Fish, pulses, meat and eggs’; (5) ‘Milk or milk products’; and (6) ‘Nuts and oil seeds’. NCD-relevant food/beverage categories and items addressed in the wider SLFBDG document are also included in the survey: (7) ‘Sweetened drinks, sweets and desserts’; (8) ‘Fast food’; (9) ‘Salt’; and (10) ‘Tea and coffee’. Within each of these categories, commonly consumed foods and beverages were defined by consulting the SLFBDGs, SLFFQ, and local nutrition researchers. The SLFBDGs provide numerical daily recommendations in the form of serving sizes for food groups 1 to 6 and salt intake (< 5 g (1 tsp)/day) and general intake advice (non-numerical) for food and beverage categories 7, 8, and 10. This makes it possible for the SLBDS to assess adherence to dietary guideline recommendations. The SLBDS, available in English, Sinhala, and Tamil, is one A4 sheet in size. It asks 37 questions about the amount of food and beverages consumed in prescribed quantities (for example, number of whole eggs); 17 yes/no diet-related questions (for example, ‘did you eat Western fast food yesterday?’); and two open-ended questions that probe for information on special diets (for example, vegetarian diet) and atypical intake pertaining to participant consumption over the past 24-h period. The survey also records respondent age, ethnicity, sex, and place of residence.

We believe that adding this fit for purpose tool to the menu of choices available to researchers will only continue to facilitate the formulation and implementation of effective and responsive healthy diet promotion interventions for Sri Lankan adults.

## Methods

**Fig. 1:**
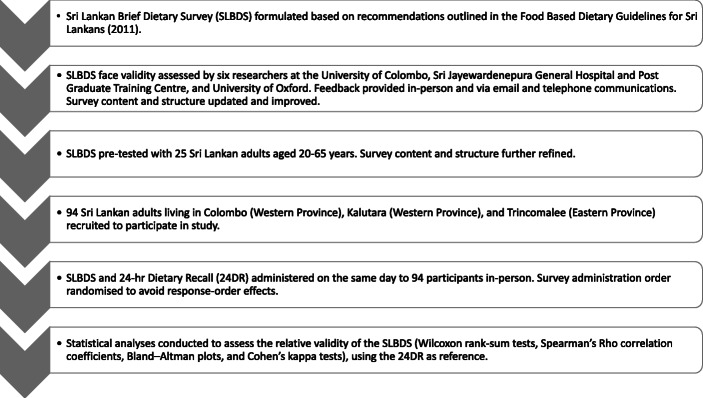
Relative validation process

Figure 1 provides an overview of the SLBDS relative validation process, from face validation and pre-testing to survey administration and statistical analyses. These steps are outlined in further detail below.

### Face validity and pre-testing

Face validity, a process whereby the SLBDS was subjectively viewed as covering what it purported to measure, was assessed by six researchers at the University of Colombo, Sri Jayewardenepura General Hospital and Post Graduate Training Centre, and University of Oxford. This process was conducted in-person and via email and telephone interviews. The feedback and discussion generated through this process focussed on prescribed SLBDS intake units (for example, coconut spoons vs cups vs tablespoons) and how best to measure notoriously difficult to capture oil, salt, and coconut intake, with researchers divided on whether to measure intake frequency (i.e. number of times item consumed in a 24-h period) or intake amount. In the latter case, we decided to record both. Clarity of survey wording and format was further refined through pre-testing with 25 Sri Lankan adults aged 20 to 65 known to our research network.

### Criterion validity testing (relative validation)

The face validated and pre-tested version of the SLBDS was subsequently tested against the 24DR to determine criterion-related validity – i.e. how well the new SLBDS estimated 24-h food intake and adherence to the SLFBDGs compared to the reference method (24DR). We selected this reference method because of its use in prior brief instrument validation studies and its statistically significant comparability to the ‘gold standard’ self-report 7-day weighed-intake dietary record (7DWR) in the target population [19]. The 24DR has a similar objective to the SLBDS and measures intake over the same time frame (24 h) whilst differing in its reliance on memory and portion size reporting format. These similarities and differences make the 24DR an appropriate reference method for this study [7, 20].

#### Study sample

Between December 2018 and February 2019, we administered both the SLBDS and the 24DR to a sample of 94 Sri Lankan adults living in urban Colombo, and urban and rural sectors in Kalutara and Trincomalee. The Trincomalee district is located in the majority Moor and Muslim Eastern Province, whereas both Colombo and Kalutara are majority Sinhalese and Buddhist Western provinces. A sample size calculation was conducted to determine the sample size required to detect a low correlation between results from the test and reference surveys (r = 0.3) with alpha and beta set to 95 and 80% respectively, and accounting for a 10% participant dropout rate. Divisional Secretaries of Colombo, Kalutara, and Trincomalee were contacted to obtain electoral lists for each Grama Niladhari Division (GND) within the district and gain permission to visit individual households for data collection. Adults aged 18 years or older who were willing and able to provide informed consent were eligible for inclusion. Data collection started at a randomly selected location within the district. From that starting point, the nearest house appearing in the relevant electoral list was approached. If a consenting adult was present, the third house to the left was approached for the next interview, and so on. If more than one eligible adult was present in the household, the interviewee was selected by drawing lots. If an eligible adult was not present or did not consent, the house next door was approached. This recruitment method was followed until 56, 29, and nine participants in Colombo, Kalutara, and Trincomalee, respectively, were recruited.

#### Dietary assessment

Each participant completed two consecutive face-to-face interviewer-administered dietary surveys, the SLBDS (new tool) and a 24DR (reference method), in the participant’s preferred language: Sinhala, Tamil or English. As the surveys were administered one after the other during the same visit, administration order was randomised to avoid response-order effects. Data collection was undertaken in participants’ homes by two experienced female Sri Lankan researchers, with 50% of the study sample randomly allocated to each interviewer (i.e. the same interviewer applied both dietary surveys). Data collectors were consulted during the survey development phase and trained through role play and practice interviews with members of the research team to deliver the surveys uniformly, closely following the provided templates and corresponding instructions.

Both surveys are structured dietary assessment tools that ask participants to recall their food and beverage consumption during the previous 24 h – a short recall period that has proven useful for minimising recall bias [21]. The two surveys differ in length, degree of survey structure, memory requirements, recall process, detail captured, and analysis burden (Table 1). The same standard household utensils were referenced to obtain information on portion sizes in both surveys and each queried whether or not the day being recalled was typical of participants' usual food intake, with interviewers prompting atypical responses for further explanation. Vegetarian status and special diets were also recorded on the paper-based templates provided.

**Table 1:** Comparison of Sri Lankan Brief Dietary Survey and 24-h Dietary Recall characteristics

Dietary assessment characteristics	Sri Lankan Brief Dietary Survey (SLBDS)	24-h Dietary Recall (24DR)
Study design	Cross-sectional	Cross-sectional
Time period of interest	Previous 24 h	Previous 24 h
Information queried	Specific components of diet (excluding contextual details)	Total diet (including contextual details)
Survey structure and length	Structured 1 A4 page Reporting units prescribed	Structured Variable length Reporting units not prescribed
Time required to complete	5–15 min	30 min – > 1 h
Recall requirements	Total consumption by food group in prescribed units	Consumption as recalled
Data coding and analysis	Data coding and unit conversion not required	Data coding and unit conversion required

#### Administration of the Sri Lankan Brief Dietary Survey

Using the SLBDS, interviewers asked participants if they had consumed each of the following food groups: (1) ‘Rice, bread, other cereals and yams’; (2) ‘Fruit’; (3) ‘Vegetables’; (4) ‘Fish, pulses, meat and eggs’; (5) ‘Milk or milk products’; (6) ‘Nuts and oil seeds’; as well as (7) ‘Sweetened drinks, sweets and desserts’; (8) ‘Fast food’; (9) ‘Salt’; and (10) ‘Tea and coffee’ in the past 24 h, and if ‘yes’, what portions (in prescribed units) of specific foods (also prescribed) within each group they consumed. After a first pass of the survey, the interviewer revisited unanswered questions.

#### Administration of the 24-h dietary recall

Detailed food and beverage consumption information was collected from participants using the 24DR method. Uninterrupted and in their own words, participants were asked to list everything they had consumed, including food and drink items and their corresponding quantities, the previous day (from waking to evening). The interviewer then probed this list for additional information: consumption time and location; item brand; further item description; and leftovers. To conclude the interview, respondents were given a further opportunity to provide additional information/detail on their 24-h intake.

#### Statistical analyses

All 188 surveys were verbatim transcribed, translated into English, and entered into Excel with 24DR data hand coded and summed to reflect SLBDS food groups/categories (1 to 10 listed above). Coding was blinded to the results of the SLBDS to avoid bias. This was achieved by coding the 24DR survey results before the researcher responsible for analysis gained access to SLBDS data. We used a chi-square test to determine whether participant characteristics differed by participant sex. As most of the dietary data were not normally distributed, we calculated the median and interquartile range (IQR) for intake of each food group and food/beverage item (based on serving size where specified in the SLFBDGs and portion size where unspecified) reported in the SLBDS and 24DR. Wilcoxon rank-sum tests were used to assess the statistical differences between medians. Correlation (r) between individual intakes collected by each measure was determined using Spearman’s Rho tests. The use of these non-parametric tests ensures that spikes at ‘zero consumption’ do not invalidate statistical assumptions. To detect differences and bias between the two methods, differences were plotted against means in Bland-Altman plots. We estimated Cohen’s kappa (k) with 95% confidence intervals to measure the inter-rater reliability for comparing achievement of recommended food group intake (where 0 = not achieved and 1 = achieved) based on the SLFBDGs between the new and reference method. For yes/no SLBDS questions: “Did you consume Western or local fast food yesterday?” and “Did you add salt, sauce/ketchup or chutney/chilli paste to your breakfast/lunch/dinner/snack?”, ‘yes’ responses were assigned a score of 1 and ‘no’ a zero. 24DR data were coded similarly: we assigned reporting of fast food (local and Western) and salt intake at specified meal times (breakfast, lunch, dinner, and snack) a score of 1 (if intake was reported) and 0 (if no intake was reported). The unweighted kappa statistic describes the level of agreement over and above chance agreement between the two measures as slight (0–0.20), fair (0.21–0.40), moderate (0.41–0.60), substantial (0.61–0.80), and almost perfect (> 0.81) [22]. To assess whether the SLBDS was an equally valid measure of dietary intake for both female and male participants, we calculated the results for each of these agreement analyses separately in females and males as a secondary analysis. We considered a *p* value < 0.05 as evidence against the null hypothesis. All statistical analyses were conducted in R version 4.0.1.

### Ethical approval

Ethics approval for this study was received from the University of Colombo (Faculty of Medicine) and the University of Oxford (Oxford Tropical Research Ethics Committee). Written informed consent was obtained from each participant prior to data collection. Compensation for participation was not provided.

## Results

Ninety-four Sri Lankan adults, including 62 females and 32 males aged 18 to 65 (mean = 40.7, SD = 12.6), agreed to participate in the study. Compared to female participants, male participants were more likely to report their dietary intake over the previous 24 h as atypical. There were no statistically significant differences between females and males with respect to age, ethnicity, place of residence, vegetarian status, and adherence to special diets. All 94 participants completed the two dietary surveys, in varying degrees of detail, and were included in analyses. SLBDS and 24DR administration time ranged from five to 15 min and 30 min to over an hour, respectively. Participant characteristics are described in Table 2.

**Table 2:** Participant characteristics

Characteristic	Females (*n* = 62)	Males (*n* = 32)	*p* value
	n		
Age group (in years)			0.39
18–29	16	6	
30–44	21	10	
45–59	18	13	
60+	5	2	
Information not provided	2	1	
Ethnicity (self-defined)			0.29
Muslim	5	9	
Sinhala	54	27	
Tamil	3	4	
District			0.33
Colombo	39	17	
Kalutara	19	10	
Trincomalee	4	5	
Sector			0.70
Urban	45	22	
Rural	17	10	
Estate	0	0	
Vegetarian			0.63
Yes	1	1	
No	61	31	
Adheres to special diet			0.21
Yes	2	3	
No	60	29	
Recall period reported as ‘typical’			0.01
Yes	56	24	
No	3	7	
Information not provided	3	1	

Table 3 presents the median and interquartile range (IQR) for the number of servings consumed for each of the six main food groups of the SLFBDGs as recorded by the SLBDS and 24DR. Median (IQR) ‘Fish, pulses, meat and eggs’ intake from the SLBDS (3.3 (2.0–5.0) daily servings) was the only food group that was significantly underestimated (*p* <  0.05) when compared to corresponding values recorded by the 24DR (4.0 (3.0–5.6) daily servings). For all other SLFBDG food groups (Table 3) and food/beverage items that do not carry a numerical daily intake recommendation in the guidelines (Table 4), there was no evidence of difference between data collected by the SLBDS and 24DR methods.

**Table 3:** Median (IQR) for the number of servings consumed for six food groups as recorded by the SLBDS and 24DR

Food group	Recommended daily servings	Daily servings (SLBDS)	Daily servings (24DR)	*p* value
		Median (IQR)	Median (IQR)	
Rice, bread, other cereals and yams	**6–11**	6.0 (4.0–8.0)	6.3 (4.3–8.9)	0.38
Fruit	**2–3**	1.0 (0.0–1.2)	0.0 (0.0–1.0)	0.17
Vegetables	**3–5**	1.0 (0.3–1.7)	1.7 (0.7–1.8)	0.09
Fish, pulses, meat and eggs	**3–4**	3.3 (2.0–5.0)	4.0 (3.0–5.6)	0.03
Milk and dairy products	**1–2**	1.0 (0.3–1.5)	1.0 (0.3–1.4)	0.46
Nuts^a^	**2–4**	0.0 (0.0–0.0)	0.0 (0.0–0.0)	1.00

^a^The unstructured 24DR was ill-equipped to collect information on the *amount* of oil intake and none of the participants volunteered this information. Median (IQR) oil consumption could therefore not be compared between the two tools

**Table 4:** Median (IQR) for the number of portions consumed for six food and beverage items as recorded by the SLBDS and 24DR

Food/beverage	Guideline recommendation	Daily servings (SLBDS)	Daily servings (24DR)	*p* value
		Median (IQR)	Median (IQR)	
Desserts	**Take less sugar, sweets or sweetened drinks.**	0.0 (0.0–0.0)	0.0 (0.0–0.0)	1.00
Sweets		0.0 (0.0–1.0)	0.0 (0.0–1.0)	0.88
Sweet fizzy drinks		0.0 (0.0–0.0)	0.0 (0.0–0.0)	0.24
Jaggery		0.0 (0.0–0.0)	0.0 (0.0–0.0)	0.69
Sugar		2.0 (0.0–4.0)	2.0 (0.0–4.0)	0.82
Tea/coffee	**Tea without milk and sugar has certain advantages. It is advisable to avoid tea/coffee closer to a main meal, as it will reduce iron absorption.**	2.0 (1.0–2.8)	2.0 (1.0–2.0)	0.76

The open-response format of the 24DR meant that participants were able to report the consumption of items that were not included in the SLBDS. These items included buns and bakery products; butter; Milo milk packets; Nestomalt/Viva/Sustagen; savoury biscuits; soya meat; and alcohol. Alcohol was the only example of an item not queried in the SLBDS for which two participants offered consumption data because they thought *“it was important for researchers to know this information in the context of health-related research” (Male participant, Colombo).*

Spearman’s Rho scores ranged at the food group level from 0.73 (‘Vegetables’) to 0.84 (‘Milk and dairy products’) and from 0.40 (‘curd’) to 1.00 (‘dosa’, ‘hoppers’, ‘milk rice’, ‘dried fish’) for specific food and beverages (Table 5). Eighty-five percent of surveyed food items showed correlations > 0.70, with three of the 34 items showing correlations between 0.56 and 0.69 (‘pasta/noodles’, ‘cooked vegetables’, and ‘cheese’) and two having correlations of 0.40 (‘curd’) and 0.43 (‘yoghurt’), both of which were underreported in the 24DR. These results indicate strong validity at the food group level for the SLBDS and for 85% of individual items, and moderate and low validity for nine and 6 % of items, respectively. Sex-disaggregated results show that correlations for ‘pasta/noodles’ (female: r = 0.38, male: r = 1.00), ‘pieces of large fruit’ (0.68, 0.85), ‘medium size sweets’ (0.60, 0.95), and ‘sweet fizzy drinks’ (0.67, 0.93) were weak or moderate in females compared to strong for males in those same categories. Correlations for ‘cooked vegetables’ (0.74, 0.65), ‘raw vegetables’ (0.83, 0.50), and ‘whole eggs’ (0.97, 0.68) were moderate in males compared to strong correlations reported for females. Only one food group, ‘Vegetables’ (0.82, 0.56), showed a comparatively weaker correlation for male than female participants.

**Table 5:** Spearman’s Rho between mean daily servings of food groups and specified foods recorded by the SLBDS and 24DR

Food groups and specified food and beverages	Total (*N* = 94)		Females (*n* = 62)		Males (*n* = 32)	
	Spearman’s ***r*** value	***p*** value	Spearman’s ***r*** value	***p*** value	Spearman’s ***r*** value	***p*** value
Rice, bread, other cereals and yams	0.82	< 0.001	0.81	< 0.001	0.79	< 0.001
Bread	0.82	< 0.001	0.87	< 0.001	0.73	< 0.001
Dosa	1.00	< 0.001	1.00	< 0.001	1.00	< 0.001
Hoppers	1.00	< 0.001	1.00	< 0.001	1.00	< 0.001
Milk rice^a^	1.00	< 0.001	1.00	< 0.001	–	–
Pasta/noodles	0.56	< 0.001	0.38	0.003	1.00	< 0.001
Pittu	0.82	< 0.001	0.71	< 0.001	1.00	< 0.001
Rice	0.93	< 0.001	0.93	< 0.001	0.86	< 0.001
Roti	0.93	< 0.001	0.95	< 0.001	0.90	< 0.001
String hoppers	0.87	< 0.001	0.86	< 0.001	0.89	< 0.001
Yam/potato/jackfruit/ breadfruit	0.82	< 0.001	0.80	< 0.001	0.83	< 0.001
Fruit	0.81	< 0.001	0.81	< 0.001	0.79	< 0.001
Fruit juice	0.94	< 0.001	0.93	< 0.001	1.00	< 0.001
Fruit salad^a^	0.70	< 0.001	0.70	< 0.001	–	–
Medium size fruit	0.87	< 0.001	0.90	< 0.001	0.79	< 0.001
Pieces large fruit	0.75	< 0.001	0.68	< 0.001	0.85	< 0.001
Vegetables	0.73	< 0.001	0.82	< 0.001	0.56	< 0.001
Cooked vegetables	0.69	< 0.001	0.74	< 0.001	0.65	< 0.001
Raw vegetables	0.74	< 0.001	0.83	< 0.001	0.50	0.003
Fish, pulses, meat and eggs	0.83	< 0.001	0.83	< 0.001	0.77	< 0.001
Cooked pulses	0.73	< 0.001	0.71	< 0.001	0.75	< 0.001
Dried fish	1.00	< 0.001	1.00	< 0.001	1.00	< 0.001
Dry sprat	0.75	< 0.001	0.77	< 0.001	0.79	< 0.001
Meat/poultry/fresh fish	0.86	< 0.001	0.85	< 0.001	0.85	< 0.001
Whole eggs	0.86	< 0.001	0.97	< 0.001	0.68	< 0.001
Milk and dairy products	0.84	< 0.001	0.86	< 0.001	0.82	< 0.001
Cheese	0.66	< 0.001	0.70	< 0.001	0.72	< 0.001
Curd^a^	0.40	< 0.001	0.39	0.002	–	–
Fresh milk	0.86	< 0.001	0.77	< 0.001	1.00	< 0.001
Milk powder	0.91	< 0.001	0.93	< 0.001	0.88	< 0.001
Yoghurt^a^	0.43	< 0.001	0.51	< 0.001	–	–
Nuts and oil seeds						
Nuts	0.83	< 0.001	0.83	< 0.001	0.82	< 0.001
Other						
Medium size desserts	0.85	< 0.001	0.80	< 0.001	1.00	< 0.001
Medium size sweets	0.73	< 0.001	0.60	< 0.001	0.95	< 0.001
Sweet fizzy drinks	0.78	< 0.001	0.67	< 0.001	0.93	< 0.001
Jaggery^a^	0.85	< 0.001	0.84	< 0.001	–	–
Sugar	0.88	< 0.001	0.86	< 0.001	0.94	< 0.001
Tea/coffee	0.86	< 0.001	0.83	< 0.001	0.91	< 0.001

^a^Correlation estimates for ‘milk rice’, ‘fruit salad’, ‘curd’, ‘yoghurt’, and ‘jaggery’ intake in males could not be calculated because there was no variance in the sample

**Fig. 2:**
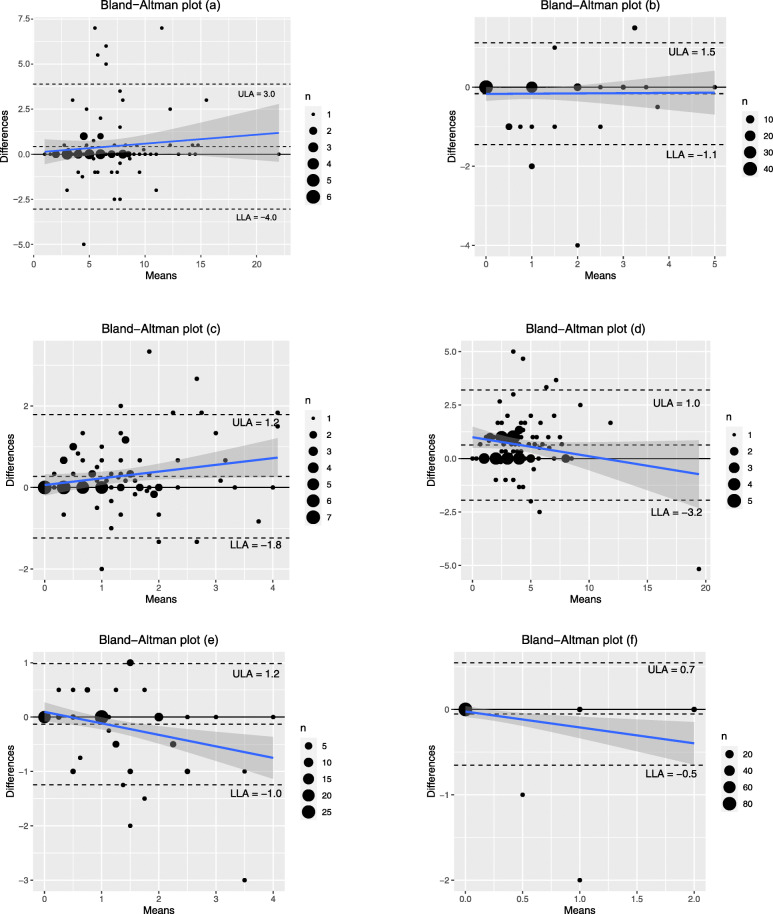
Bland-Altman plots for (**a**) Rice, bread, other cereals and yams; (**b**) Fruit; (**c**) Vegetables; (**d**) Fish, pulses, meat and eggs; (**e**) Milk and dairy products; (**f**) Nuts. The size of data points is proportional to the number of observations at each value. ULA = upper limit of agreement; LLA = lower limit of agreement

Visual exploration of agreement between the SLBDS and 24DR using Bland-Altman plots (Fig. 2) showed that for ‘Rice, bread, other cereals and yams’, there is minimal difference between the intake recorded on the tested method compared to the reference method between 0 and 10 servings. At > 10 servings, the SLDBS tended to overestimate recorded intake compared with the reference method. ‘Vegetable’ intake was similarly measured by the two methods, with the SLBDS tending to slightly overestimate intake as the number of servings consumed increased. There was relatively little variation across all levels of intake for ‘Fruit’, showing acceptable agreement between the two methods. ‘Fish, pulses, meat and eggs’, ‘Milk and dairy products’, and ‘Nuts’ all tended to be slightly underestimated by the SLBDS compared to the 24DR as the number of servings consumed increased. For each category, less than 6% of participants fell outside of the limits of agreement (LA).

Table 6 presents kappa (k) statistics and 95% confidence intervals for agreement between participant achievement of the SLFBDG recommendations calculated from the SLBDS and 24DR. k values range from 0.59 (95% CI: 0.32–0.86) for ‘Vegetables’ to 0.81 (95% CI: 0.66–0.96) for ‘Fruit’ indicating a moderate to strong level of agreement. Sex disaggregated kappa statistics (Table S2) do not indicate statistically significant sex-based differences.

**Table 6:** Cohen’s kappa for agreement between achievement of the Sri Lankan Food Based Dietary Guideline recommendations calculated from the SLBDS and 24DR

Food groups	Total (*N* = 94)		
	% participants who achieved SLFBDG recommendation (95% CI)		***κ*** value (95% CI)
	SLBDS	24DR	
Rice, bread, other cereals and yams	52.1 (42.0, 62.2)	57.5 (47.5, 67.4)	0.76 (0.63, 0.89)
Fruit	23.40 (14.9, 32.0)	19.2 (11.2, 27.1)	0.81 (0.66, 0.96)
Vegetables	7.5 (2.3, 12.8)	12.8 (6.0, 19.5)	0.59 (0.32, 0.86)
Fish, pulses, meat and eggs	64.9 (55.3, 74.6)	78.7 (70.5, 87.0)	0.61 (0.45, 0.78)
Milk and dairy products	34.0 (24.5, 43.7)	30.9 (21.5, 40.2)	0.64 (0.47, 0.80)

Table 7 presents kappa (k) statistics and 95% confidence intervals for food and beverage items (‘medium size desserts’; ‘medium size sweets’; ‘sweet fizzy drinks’; ‘sugar’; ‘jaggery’; ‘tea/coffee’) surveyed in the SLBDS for which numerical intake recommendations are not outlined in the SLFBDGs. We assigned consumption data in both the SLBDS and 24DR a score of 0 (if an intake of zero portions was reported) or 1 (if reported intake was > 0 portions) and calculated the kappa statistic for each. The calculated k values range from 0.71 (95% CI: 0.56–0.86) for ‘medium size sweets’ to 0.85 for both ‘medium size desserts’ (95% CI: 0.71–0.99) and ‘jaggery’ (95% CI: 0.65–0.92) indicating a strong level of agreement between the number of participants reporting consumption > 0 for the six food items listed above in both the SLBDS and 24DR. Sex-disaggregated kappa statistics (Table S3) show no evidence for sex-based differences in agreement.

**Table 7:** Cohen’s kappa for agreement between number of participants reporting consumption > 0 in both the SLBDS and 24DR

Food and beverage item	Total (*N* = 94)		
	% participants that reported consumption > 0 servings (95% CI)		***κ*** value (95% CI)
	SLBDS	24DR	
Medium size desserts	17.0 (9.4, 24.6)	17.0 (9.4, 24.6)	0.85 (0.71, 0.99)
Medium size sweets	34.4 (24.5, 43.6)	31.9 (22.5, 41.3)	0.71 (0.56, 0.86)
Sweet fizzy drinks	18.1 (10.3, 25.9)	11.7 (5.2, 18.2)	0.75 (0.56, 0.94)
Jaggery	4.3 (0.2, 8.3)	3.2 (0.0, 6.7)	0.85 (0.57, 1.00)
Sugar	66.0 (56.4, 75.5)	69.2 (59.8, 78.5)	0.78 (0.65, 0.92)
Tea/coffee	82.0 (74.2, 89.8)	83.0 (75.4, 90.6)	0.74 (0.56, 0.92)

Table 8 presents kappa (k) statistics and 95% confidence intervals for yes/no SLBDS questions: “Did you consume Western or local fast food yesterday?” and “Did you add salt, sauce/ketchup or chutney/chilli paste to your breakfast/lunch/dinner/snack?”. A strong level of agreement between fast food reporting in the SLBDS and 24DR is indicated by high k values: 0.80 (95% CI: 0.68–0.93) for ‘Fast food’ (any type); 0.81 (95% CI: 0.68–0.95) for ‘local’; and 0.79 (95% CI: 0.64–0.94) for ‘Western’. Sex-disaggregated k values do not suggest statistically significant differences in fast food reporting on the two surveys between females and males (Table S4). K values for reporting of salt at different meal times are low, ranging from 0.03 to 0.09, indicating slight agreement between the number of participants reporting their salt consumption on the SLBDS and the 24DR. There is no evidence for sex-based differences in agreement for the reporting of salt intake (Table S4).

**Table 8:** Cohen’s kappa for agreement between number of participants reporting consumption of fast food and salt intake in both the SLBDS and 24DR

Food and beverage item	Total (*N* = 94)		
	% participants that reported consumption > 0 servings (95% CI)		***κ*** value (95% CI)
	SLBDS	24DR	
Fast food	43.6 (33.6, 53.6)	42.6 (32.6, 52.6)	0.80 (0.68, 0.93)
Western	26.6 (17.7, 35.4)	19.2 (11.2, 27.1)	0.79 (0.64, 0.94)
Local	25.5 (16.7, 34.4)	28.7 (19.6, 37.9)	0.81 (0.68, 0.95)
Added salt			
Breakfast	95.7 (91.7, 99.8)	52.1 (42.0, 62.2)	0.09 (0.01, 0.18)
Lunch	95.7 (91.7, 99.8)	42.6 (32.6, 52.6)	0.06 (0.00, 0.13)
Dinner	95.7 (91.7, 99.8)	43.6 (33.6, 53.6)	0.03 (−0.04, 0.10)
Snack	96.8 (93.3, 100.0)	27.7 (18.6, 36.7)	0.03 (0.00, 0.05)

The SLBDS specifically queried oil (method, reheating, and amount) and coconut (fresh and powder) consumption. For both oil and coconut, no intake information was reported in the 24DR for either amount consumed or whether oil used in cooking was reheated. Conversely, a greater proportion of participants specified method of cooking with oil (for example, fried, deep fried, and tempered) in the 24DR (97%) compared with the SLBDS (71%). All k values for oil and coconut reporting were low, ranging from 0.00 to 0.15 (Table S5). Sex-disaggregated k values do not indicate significant differences in oil and coconut reporting between female and male participants (Table S7).

## Discussion

The SLBDS was developed for and relatively validated with the study population in question to assess food intake and adherence to the SLFBDG recommendations for potential use as a dietary surveillance supplement and in multiple risk factor NCD studies. We have demonstrated its relative validity in comparison to a 24DR that has been previously validated against a 7DWR in the target population [19]. At the food group level, the SLBDS performed well. Correlations between intake recorded on the new and reference tool were strong (r > 0.73) and agreement between recommendation adherence calculated by the two tools was moderate to strong. At the food and beverage item level, 85% of surveyed items showed correlations > 0.7, with five of the 34 items showing correlations between 0.40 and 0.69. There was no evidence of difference in agreement between female and male participants.

It was not possible to compare salt and cooking oil intake as well as fresh and powdered coconut consumption between the two methods that were primarily intended to investigate food, and not nutrient, intake. The 24DR failed to capture any data on the amount of coconut, cooking oil, and salt consumed by participants. Difficulties and inconsistences in measuring self-reported added salt and oil intake are not unique to this study and are well documented in the literature [23–25]. This is in part due to the fact that salt and fats are often ‘hidden’ in foods; salt, oil, and coconut are not always added in exact amounts during cooking; and participants who are not involved in food preparation cannot be expected to accurately recall meal ingredients in dietary surveys. In the Sri Lankan context, capturing intake is further hindered by the absence of an updated, consensus nutritional composition database for Sri Lankan foods [26, 27]. Given nonoptimal intake of dietary fat and salt are established dietary risk factors for the development of NCDs and self-report measures are widely utilised in diet-disease research, accurate and appropriate measurement of these commonly consumed yet underreported nutrients remains a challenge [28–30]. Context-specific qualitative studies that explore how people use and consume coconut, oil, and salt, and importantly, how they report use and consumption in their own words are necessary for tool revision and improvement. Over 95% of participants recorded whether they added salt or salty sauce to meals (breakfast, lunch, dinner, and snack) when prompted by the SLBDS, as compared to 28% (snack) to 52% (breakfast) on the 24DR when asked about meal ingredients. This demonstrates the utility of the SLBDS in prompting reporting of salt intake data akin to that collected by the WHO STEPS instrument which asks participants ‘how often’ salt is added to food and cooking (with possible answers ranging from ‘Never’ to ‘Always’). However, in the absence of objective (for example, a 24-h urinary assessment) or repeat self-report measurements, we are unable to conclude which survey - the SLBDS or 24DR – reported ‘true’ intake. Multiple administrations of the SLBDS at non-consecutive time points would be required to capture daily variability and average consumption patterns – for salt and all other dietary components queried – over time. Prompting participants for information on oil reheating on the SLBDS was another feature of the SLBDS not present in the 24DR.

### Study limitations

A key limitation of this study is that it only included participants from three of 25 districts in Sri Lanka, which limits the generalisability of findings. Collecting additional demographic data, including employment status, education, income, and whether or not survey participants are involved in food preparation would provide additional information to support SLBDS validity and subsequent context-specific improvements for different subsets of the population. This study assessed the relative validity of the SLBDS but did not investigate test-retest reliability – a key performance characteristic for the selection of dietary surveillance measures that capture ‘usual intake’. Further research with a larger and more diverse study sample that tests the validity and reliability of the SLBDS against an unbiased criterion measure of true intake or multiple ‘error-prone’ reference measures (convergent validity) is therefore required before the SLBDS can be utilised widely to measure the dietary intake of the Sri Lankan adult population [31, 32]. Study districts were selected to represent a range of Sri Lankan demographics. Participants in this study, however, were low consumers of certain food items. We therefore also recommend that the SLBDS be tested in populations with different patterns of consumption (see Table S7).

### Implications of this research

Food consumption surveys provide vital information for the formulation, implementation, and evaluation of appropriate and effective diet-related policies and targeted programmes. A validated research tool that is able to assess the effectiveness of policies and programmes based on the SLFBDGs is therefore necessary to track progress towards national health and nutrition goals [33]. The SLBDS responds to this need, providing a contextually appropriate dietary surveillance measure that has clear public health relevance. Depending on the specific research question or intervention of interest (for example, a national education campaign to promote fruit and vegetable intake), single food groups that demonstrated strong correlation and agreement in the SLBDS can be queried in lieu of administering the SLBDS in its entirety or the more time and resource intensive 24DR, FFQ, and STEPS survey.

The more detailed 24DR captured intake of food/beverages not included on the SLBDS or in the SLFBDGs. This study did not assess the appropriateness or comprehensiveness of the current SLFBDGs that were last updated in 2011 or the health benefits of complying with guideline recommendations. Future research should focus on updating the SLFBDGs to continue to reflect population diets under conditions of nutrition transition and to integrate environmental sustainability considerations that contribute to the achievement of global environmental targets [34]. The health and environmental implications of adopting current and updated guideline recommendations should also be assessed – projects that might be assisted by or contribute to the continual improvement of the SLBDS.

This study supports a shift toward focussing attention on comparable surveillance outcomes (i.e. achievement of recommended daily intake for different food groups) over prioritising the consistent use of ‘off the shelf’ tools – a shift that may have implications for reducing errors associated with self-report dietary measurement methods [35]. The development of consensus portion size conversion guidelines for the Sri Lankan population would further facilitate the ability to compare study outcomes and make the survey innovation process and subsequent validation against existing tools easier for researchers.

Other dimensions of tool validation, including social validity – i.e. whether participants feel the SLBDS is an acceptable and important research tool – need to be explored through qualitative research. We would also like to explore online delivery of the SLBDS to further reduce administration and analysis burden; potential interviewer and social desirability bias; the time and cost associated with administering repeat surveys; and to support real-time translation of data for evidence-informed decision-making.

## Conclusion

Addressing the problem of suboptimal diet requires context-specific solutions informed by context-specific data collected by context-specific tools. Our study shows that the SLBDS demonstrates promise as fit for purpose research tool to supplement and support dietary surveillance activities in Sri Lanka. The next step is to assess SLBDS test-retest reliability.

## Supplementary Information

**Additional file 1: Fig. S1.** Sri Lankan Brief Dietary Survey. **Table S2.** Cohen’s kappa for agreement between achievement of the Sri Lankan Food Based Dietary Guideline recommendations calculated from the SLBDS and 24DR, by participant sex. **Table S3.** Cohen’s kappa for agreement between number of participants reporting consumption > 0 in both the SLBDS and 24DR, by participant sex. **Table S4.** Cohen’s kappa for agreement between number of participants reporting consumption of fast food and salt intake in both the SLBDS and 24DR, by participant sex. **Table S5.** Cohen’s kappa for agreement between number of participants reporting cooking oil and coconut consumption data in both the SLBDS and 24DR. **Table S6.** Cohen’s kappa for agreement between number of participants reporting cooking oil and coconut consumption data in both the SLBDS and 24DR, by participant sex. **Table S7.** Number of participants that reported zero consumption on the SLBDS and 24DR.

**Additional file 2.** Consolidated criteria for reporting qualitative studies (COREQ): 32-item checklist.

## Data Availability

The results of the study are reported in the main text and the supplementary information. Ethical approval for this study does not extend to the sharing of participant data. Please contact the corresponding author for further information.

## Abbreviations

**LA**: Limits of Agreement
**LLA**: Lower Limit of Agreement
**SLBDS**: Sri Lankan Brief Dietary Survey
**SLFBDGs**: Food Based Dietary Guidelines for Sri Lankans
**SLFFQ**: Food Frequency Questionnaire for Sri Lankan Adults
**NCD**: Non-communicable disease
**ULA**: Upper Limit of Agreement
**WHO**: World Health Organization
**24DR**: 24-h Dietary Recall
