# Supplementary material for: Relative validity of a brief dietary survey to assess food intake and adherence to national dietary guidelines among Sri Lankan adults
Source: BMC Nutr. 2020 Nov 26;6:68. doi: 10.1186/s40795-020-00391-2 (PMC7690106; doi:10.1186/s40795-020-00391-2)
Supplement: Supplementary file 2 — Additional file 2. Consolidated criteria for reporting qualitative studies (COREQ): 32-item checklist. [file 40795_2020_391_MOESM2_ESM.docx]

**Consolidated criteria for reporting qualitative studies (COREQ): 32-item checklist**

*Developed from:*

*Tong A, Sainsbury P, Craig J. Consolidated criteria for reporting qualitative research (COREQ): a 32-item checklist for interviews and focus groups. International Journal for Quality in Health Care. 2007. Volume 19, Number 6: pp. 349 – 357*

| **No. Item** | **Description** | **Reported on Page #** |
| --- | --- | --- |
| **Domain 1: Research team and reﬂexivity** |  |  |
| *Personal Characteristics* |  |  |
| 1. Interviewer/facilitator | Dr Buwaneka Kalupahana (BK) and Dr Santhushya Fernando (SF) administered dietary surveys. | Methods page 9 |
| 2. Credentials | MBBS Bachelor of Medicine, Bachelor of Surgery | N/A |
| 3. Occupation | Research assistant (BK) and Researcher/Dr (SF). | Author affiliations/information page 1 |
| 4. Gender | Female | Methods page 9 |
| 5. Experience and training | BK: Research assistant and data collector for the Family Health Bureau of Sri Lanka (2017 - 2018).  SF: NCD researcher (with experience in dietary data collection) (2009 – present); Medical officer (2003 – 2011). | N/A |
| *Relationship with participants* |  |  |
| 6. Relationship established | No relationship was established with participants prior to the study. | N/A |
| 7. Participant knowledge of the interviewer | Participants were provided with a study information sheet. This included study aims and objectives, research questions, interview questions, and information about the researchers involved in the study (for example, affiliated institution, designation, and gender). The data collectors reintroduced themselves and their credentials in-person to study participants. | N/A |
| 8. Interviewer characteristics | Researcher characteristics/positionality (gender, nationality, and credentials) were communicated with participants and reported in the manuscript. | Methods page 9 |
| **Domain 2: study design** |  |  |
| *Theoretical framework* |  |  |
| 9. Methodological orientation and Theory | N/A  (Quantitative statistical analysis of dietary intake data.) | Methods page 11 |
| *Participant selection* |  |  |
| 10. Sampling | Participants were selected using purposive sampling. Divisional Secretaries of Colombo, Kalutara, and Trincomalee were contacted to obtain electoral lists for each Grama Niladhari Division (GND) within the district and gain permission to visit individual households for data collection. | Methods page 9 |
| 11. Method of approach | Participants were approached face-to-face. | Methods page 9 |
| 12. Sample size | A sample size calculation was conducted to determine the sample size required to detect a low correlation between results from the test and reference surveys (r = 0.3) with alpha and beta set to 95% and 80% respectively, and accounting for a 10 percent participant dropout rate. 94 households were invited to participate in the study. | Methods page 9 |
| 13. Non-participation | None. Of the 94 households approached to participate, 94 residing adults provided written informed consent to participant. | Results page 12 |
| *Setting* |  |  |
| 14. Setting of data collection | Data were collected in participants’ homes. | Methods page 9 |
| 15. Presence of non-participants | Non-participants were not present during data collection. | N/A |
| 16. Description of sample | Reported characteristics of the sample included participant gender, age, self-defined ethnicity, vegetarian status, adherence to special diet (Y/N), and place of residence. | Results page 12 |
| *Data collection* |  |  |
| 17. Interview guide | N/A  (Two dietary intake surveys, one novel and one reference, were administered by researchers.) | Methods page 9 |
| 18. Repeat interviews | N/A  (Each participant responded to two different dietary intake surveys during the same interview.) | Methods page 9 |
| 19. Audio/visual recording | N/A  (Researchers recorded the participant’s dietary intake on printed survey templates.) | Methods page 9-10 |
| 20. Field notes | N/A | N/A |
| 21. Duration | Interviewer-administered surveys ranged from 15 minutes to one hour in duration. | Results page 12 |
| 22. Data saturation | N/A | N/A |
| 23. Transcripts returned | N/A  (Following best practice, dietary surveys were multiple pass.) | Methods page 11 |
| **Domain 3: analysis and ﬁndings** |  |  |
| *Data analysis* |  |  |
| 24. Number of data coders | N/A  (One researcher undertook quantitative data analysis under supervision of the wider research team.) | Methods page 11 |
| 25. Description of the coding tree | N/A | N/A |
| 26. Derivation of themes | N/A | N/A |
| 27. Software | N/A  (All statistical analyses were conducted in R version 4.0.1.) | Methods page 12 |
| 28. Participant checking | N/A | N/A |
| *Reporting* |  |  |
| 29. Quotations presented | N/A | N/A |
| 30. Data and ﬁndings consistent | The authors believe that there is consistency between data presented (in the results section) and study findings (outlined in the discussion section). | Results and discussion |
| 31. Clarity of major themes | N/A | N/A |
| 32. Clarity of minor themes | N/A | N/A |
