# Supplementary material for: Relative validity of a brief dietary survey to assess food intake and adherence to national dietary guidelines among Sri Lankan adults
Source: BMC Nutr. 2020 Nov 26;6:68. doi: 10.1186/s40795-020-00391-2 (PMC7690106; doi:10.1186/s40795-020-00391-2)
Supplement: Supplementary file 3 [file 40795_2020_391_MOESM1_ESM.docx]

Supplementary materials 

Figure S1. Sri Lankan Brief Dietary Survey

| About you: | Sex: | Age: | Grama Niladari division: | Are you a vegetarian? Yes / No Are you on a special diet?  Yes / No If yes, what is the diet name: |
| --- | --- | --- | --- | --- |
| Ethnicity: |
| What was the day yesterday? (Please circle)                 Monday / Tuesday / Wednesday / Thursday / Friday / Saturday / Sunday |
| Please tell us about what you ate yesterday… |
| Name of food group | Did you consume this food group? (Please circle) | How much did you consume? [1 cup = 200ml tea cup] |
| Rice, bread, other cereals and yams | Yes / No | Number of coconut spoons of rice = ______ Number of pieces of milk rice = ______ Number of bread slices =______ Number of hoppers = ______ and string hoppers = ______ Number of roti = ______ , dosa = ______ and pittu = ______ Number of coconut spoons of pasta/noodles = ______ Number of coconut spoons of yams/potatos/jackfruit = ______ |
| Fruits | Yes / No | Number of medium size fruit  = ______ Number of pieces of large fruit = ______ Number of cups of fruit salad =______ Number of glasses of fresh fruit juice = ______ |
| Vegetables | Yes / No | Number of tablespoons of cooked vegetables =______ Number of tablespoons of dark green leaves (Mallums) =______ |
| Fish, pulses, meat and eggs | Yes / No | Number of pieces of meat/poultry/fresh fish = ______ (1 piece is approximately the size of a small matchbox) Number of coconut spoons of cooked pulses =______ (Examples: Chickpea, green gram, cowpea, soy bean, ulundu, lentil) Number of whole eggs =______ Pieces/small squares of dried fish = ______ |
| Milk or milk products | Yes / No | Number of cups of fresh milk = ______ Number of cups of yoghurt = ______ Number of cups of curd = ______ Number of teaspoons of milk powder =______ |
| Nuts and oil seeds | Yes / No | Number of handfuls of nuts = ______ Type(s) of oil added to cooking = ________________ Was this oil reheated oil?   Yes / No (If known) number of tablespoons of oil added to cooking = _______ Per household: Number of whole coconuts = ______ Number of packets of coconut powder = ______ How many people did you cook for? ______ |
| Sweetened drinks, sweets and desserts | Yes / No | Number of glasses of sweet fizzy drink = ______ Number of medium size sweets = ______ Number of medium size desserts (ice cream, pudding) = ______ |
| Fast food | Did you eat Western fast food yesterday?  Yes / No (Examples: French fries, pizza, McDonald’s, KFC, pattie, cutlet, Chinese roll, pastries) Did you eat Local fast food yesterday?  Yes / No (Examples: samosa, papadam, egg role, wadai, murukku, manioc chips) |
| Salt | Yesterday, during cooking or after, did you add salt, sauce/ketchup or chutney/chilli paste to your Breakfast   Yes / No   Lunch   Yes / No   Dinner   Yes / No   Snack   Yes / No Total pinches of salt added = ______ |
| Tea | How many cups of tea did you have yesterday? ______ How many teaspoons of sugar did you add to each cup? ______ How many pieces of Jaggery did you have with each cup? ______ |
| Would you consider yesterday a ‘typical day’ that represents the type and amount of food you usually consume? Please circle one response: Yes / No / to some extent      Please explain your response: |

Table S2. Cohen's kappa for agreement between achievement of the Sri Lankan Food Based Dietary Guideline recommendations calculated from the SLBDS and 24DR, by participant sex

| Food groups | Female (n=62) | Male (n=32) |
| --- | --- | --- |
| % female participants who achieved SLFBDG recommendation (95% CI) | κ value (95% CI) | % male participants who achieved SLFBDG recommendation (95% CI) | κ value (95% CI) |
| SLBDS | 24DR | SLBDS | 24DR |  |
| Rice, bread, other cereals and yams | 43.6  (31.2, 55.9) | 68.6 (57.2, 80.3) | 0.74 (0.57, 0.91) | 46.8 (29.5, 64.1) | 78.1 (63.8, 92.5) | 0.76 (0.51, 1.00) |
| Fruit | 17.7 (8.2, 27.3) | 34.4  (22.6, 46.2) | 0.76 (0.54, 0.98) | 14.5 (2.3, 26.7) | 28.1 (12.6, 43.7) | 0.86 (0.66, 1.00) |
| Vegetables | 6.5  (0.2, 12.6) | 9.4 (2.1, 16.6) | 0.45 (0.09, 0.81) | 12.9 (1.3, 24.5) | 12.5 (1.0, 24.0) | 0.84 (0.54, 1.00) |
| Fish, pulses, meat and eggs | 59.7 (47.5, 71.9) | 75.0 (64.2, 85.8) | 0.60  (0.40, 0.80) | 75.8 (61.0, 90.6) | 84.4 (71.8, 97.0) | 0.71 (0.42, 1.00) |
| Milk and dairy products | 35.5  (23.6, 47.4) | 31.3 (19.7, 42.8) | 0.60 (0.40, 0.80) | 33.9 (17.5, 50.3) | 25.0 (10.0, 40.0) | 0.69 (0.42, 0.97) |

Table S3. Cohen's kappa for agreement between number of participants reporting consumption > 0 in both the SLBDS and 24DR, by participant sex	

| Food and beverage item | Female (n=62) | Male (n=32) |
| --- | --- | --- |
| % female participants that reported consumption > 0 servings (95% CI) | κ value (95% CI) | % male participants that reported consumption > 0 servings (95% CI) | κ value (95% CI) |
| SLBDS | 24DR | SLBDS | 24DR |
| Medium size desserts | 19.4 (9.5, 29.2) | 19.4 (9.5, 29.2) | 0.8 (0.6, 1.0) | 12.5 (1.0, 24.0) | 12.5 (1.0, 24.0) | 1.00 (1.00, 1.00) |
| Medium size sweets | 32.3 (20.6, 43.9) | 30.7 (19.2, 42.1) | 0.6 (0.4, 0.8) | 37.5 (20.7, 54.3) | 34.4 (17.9, 50.8) | 0.93 (0.80, 1.00) |
| Sweet fizzy drinks | 16.1 (7.0, 25.3) | 8.1 (1.3, 14.8) | 0.6 (0.3, 0.9) | 21.9 (7.6, 36.2) | 18.8 (5.2, 32.3) | 0.90 (0.72, 1.00) |
| Jaggery | 6.5 (0.3, 12.6) | 4.8 (0.0, 10.2) | 0.9 (0.6, 1.0) | 0.0 (0.0, 0.0) | 0.0 (0.0, 0.0) | - |
| Sugar | 61.3 (49.2, 73.4) | 69.4 (57.9, 80.8) | 0.8 (0.6, 0.9) | 75.0 (60.0, 90.0) | 68.8 (52.7, 84.8) | 0.85 (0.64, 1.00) |
| Tea/coffee | 77.4 (67.0, 87.8) | 82.3 (72.8, 91.8) | 0.8 (0.5, 1.0) | 90.6 (80.5, 100.0) | 84.4 (71.8, 97.0) | 0.72 (0.35, 1.00) |

 

Table S4. Cohen's kappa for agreement between number of participants reporting consumption of fast food and salt intake in both the SLBDS and 24DR, by participant sex

| Food and beverage item | Female (n=62) | Male (n=32) |
| --- | --- | --- |
| % female participants that reported consumption > 0 servings (95% CI) | κ value  (95% CI) | % male participants that reported consumption > 0 servings (95% CI) | κ value (95% CI) |
| SLBDS | 24DR | SLBDS | 24DR |
| Fast food | 38.7 (26.6, 50.8) | 53.1 (40.7, 65.6) | 0.76 (0.59, 0.93) | 37.1 (20.4, 53.8) | 53.1 (35.8, 70.4) | 0.87 (0.71, 1.00) |
| Western | 21.0 (10.8, 31.1) | 12.9 (4.6, 21.2) | 0.72 (0.49, 0.950 | 12.0 (0.7, 23.3) | 10.0 (-0.4, 20.4) | 0.86 (0.68, 1.00) |
| Local | 15.0 (6.1, 23.9) | 18.0 (8.4, 27.6) | 0.79 (0.62, 0.97) | 9.0 (-0.9, 18.9) | 9.0 (-0.9, 18.9) | 0.85 (0.64, 1.00) |
| Added salt |  |  |  |  |  |  |
| Breakfast | 93.6 (87.4, 99.7) | 100.0 (100.0, 100.0) | 0.15 (0.01, 0.28) | 53.2 (35.9, 70.5) | 50.0 (32.7, 67.3) | 0.00 (0.00, 0.00) |
| Lunch | 93.6 (87.4, 99.7) | 100.0 (100.0, 100.0) | 0.08 (0.00, 0.17) | 38.7 (21.8, 55.6) | 50.0 (32.7, 67.3) | 0.00  (0.00, 0.00) |
| Dinner | 93.6 (87.4, 99.7) | 100.0 (100.0, 100.0) | 0.04 (-0.06, 0.14) | 41.9 (24.8, 59.0) | 46.9 (29.6, 64.2) | 0.00 (0.00, 0.00) |
| Snack | 95.2 (89.8, 100.0) | 100.0 (100.0, 100.0) | 0.04 (-0.01, 0.10) | 30.7 (14.7, 46.6) | 21.9 (07.6, 36.2) | 0.00 (0.00, 0.00) |

Table S5. Cohen's kappa for agreement between number of participants reporting cooking oil and coconut consumption data in both the SLBDS and 24DR

|  | Total (N=94) |
| --- | --- |
|  | % participants that reported consumption data (95% CI) | κ value (95% CI) |
|  | SLBDS | 24DR |
| Oil (used in cooking) |
| Amount | 70.2 (61.0, 79.5) | 0.0 (0.0, 0.0) | 0.00 (0.00, 0.00) |
| Method | 71.3 (62.1, 80.4) | 96.8 (93.3, 100.0) | 0.15 (-0.00, 0.31) |
| Reheated | 80.6 (72.9, 88.8) | 0.0 (0.0, 0.0) | 0.00 (0.00, 0.00) |
| Coconut(s) (used in cooking) |
| Whole coconut(s) | 87.2 (80.5, 94.0) | 0.0 (0.0, 0.0) | 0.00 (-3.5e-10, 3.5e-10) |
| Coconut powder | 92.6 (87.3, 97.9) | 0.0 (0.0, 0.0) | 0.00 (0.00, 0.00) |

Table S6. Cohen's kappa for agreement between number of participants reporting cooking oil and coconut consumption data in both the SLBDS and 24DR, by participant sex

|  | Female (n=62) | Male (n=32) |
| --- | --- | --- |
|  | % female participants that reported consumption data (95% CI) | κ value (95% CI) | % male participants that reported consumption data (95% CI) | κ value (95% CI) |
|  | SLBDS | 24DR | SLBDS | 24DR |
| Oil (used in cooking) |
| Amount | 83.9 (74.1, 93.0) | 0.0 (0.0, 0.0) | 0.0 (0.0, 0.0) | 43.8 (26.6, 60.9) | 0.0 (0.0, 0.0) | 0.0 (0.0, 0.0) |
| Method | 67.7 (56.1, 79.4) | 98.4  (95.3, 100.0) | 0.1 (-0.1, 0.2) | 78.1 (63.8, 92.5) | 93.8 (85.4, 100.0) | 0.38  (-0.01, 0.78) |
| Reheated | 87.1 (78.8, 95.4) | 0.0 (0.0, 0.0) | 0.0 (0.0, 0.0) | 68.8 (52.7, 84.8) | 0.0 (0.0, 0.0) | 0.0 (0.0, 0.0) |
| Coconut(s) (used in cooking) |
| Whole coconut(s) | 98.4 (95.3, 100.0) | 0.0 (0.0, 0.0) | 0.0 (0.0, 0.0) | 65.6 (49.2, 82.1) | 0.0 (0.0, 0.0) | 0.0 (0.0, 0.0) |
| Coconut powder | 96.8 (92.4, 100.0) | 0.0 (0.0, 0.0) | 0.0 (0.0, 0.0) | 84.4 (71.8, 97.0) | 0.0 (0.0, 0.0) | 0.0 (0.0, 0.0) |

Table S7. Number of participants that reported zero consumption on the SLBDS and 24DR

| Food and beverage item | Total (N=94) |
| --- | --- |
| Number of participants that reported zero consumption |
| SLBDS | 24DR |
| Rice, bread, other cereals and yams |
| Bread | 65.0 | 64.0 |
| Dosa | 85.0 | 85.0 |
| Hoppers | 88.0 | 88.0 |
| Milk rice | 91.0 | 91.0 |
| Pasta/noodles | 90.0 | 91.0 |
| Pittu | 92.0 | 91.0 |
| Rice | 2.0 | 1.0 |
| Roti | 78.0 | 78.0 |
| String hoppers | 76.0 | 74.0 |
| Yam/potato/jackfruit/breadfruit | 57.0 | 50.0 |
| Fruit |
| Fruit juice | 85.0 | 86.0 |
| Fruit salad | 92.0 | 93.0 |
| Medium size fruit | 54.0 | 60.0 |
| Pieces large fruit | 84.0 | 88.0 |
| Vegetables |
| Cooked vegetables | 20.0 | 17.0 |
| Raw vegetables | 53.0 | 50.0 |
| Fish, pulses, meat and eggs |
| Cooked pulses | 33.0 | 26.0 |
| Dried fish | 79.0 | 79.0 |
| Dry sprat | 84.0 | 80.0 |
| Meat/poultry/fresh fish | 18.0 | 18.0 |
| Whole eggs | 64.0 | 66.0 |
| Milk and dairy products |
| Cheese | 91.0 | 91.0 |
| Curd | 91.0 | 92.0 |
| Fresh milk | 80.0 | 81.0 |
| Milk powder | 34.0 | 33.0 |
| Yoghurt | 84.0 | 92.0 |
| Nuts and oil seeds |
| Nuts | 83.0 | 86.0 |
| Other |
| Medium size desserts | 78.0 | 78.0 |
| Medium size sweets | 62.0 | 64.0 |
| Sweet fizzy drinks | 77.0 | 83.0 |
| Jaggery | 90.0 | 91.0 |
| Sugar | 32.0 | 29.0 |
| Tea/coffee | 17.0 | 16.0 |
